# Cultivating support during COVID‐19 through clinical supervision: A discussion article

**DOI:** 10.1002/nop2.1800

**Published:** 2023-05-07

**Authors:** Claire O'Donnell, Kathleen Markey, Louise Murphy, James Turner, Owen Doody

**Affiliations:** ^1^ Department of Nursing and Midwifery, Faculty of Education and Health Sciences, Health Research Institute University of Limerick Limerick Ireland; ^2^ Department of Nursing and Midwifery, College of Health and Life Sciences Sheffield Hallam University Sheffield UK

**Keywords:** clinical supervision, clinical supports, COVID‐19, education, nurse educators, nurses, pandemic experience, student nurses

## Abstract

**Aim:**

This article aims to discuss how clinical supervision is an important approach in supporting frontline nurses and students during and post COVID‐19 through the lens of the nursing metaparadigms.

**Design:**

Discussion article.

**Methods:**

Discourse of the literature considering the importance of working collaboratively with healthcare and educational organisations in operationalising clinical supervision.

**Results:**

The evidence base supporting clinical supervision as an effective support strategy for nurses exists, however, its implementation and practice has become sporadic. A resurgence is required to support student's and nurse's during this pandemic. It is timely for nurse educators to creatively engage with clinical partners in supporting clinical supervision to enhance both nurses and students pandemic practice experiences. Clinical supervision is proposed as one strategy to support and guide both nurses and students to develop, strengthen and challenge the effectiveness of their care during COVID‐19.

## 1 INTRODUCTION

Healthcare systems globally operate in fast‐paced complex environments and the advent of COVID‐19 has added extra layers of intricacy further challenging care delivery and nurse education (Carolan et al., 2020; Martin & Snowdon, 2020). While support for nurses as frontline workers during COVID‐19 pandemic has been affirmed by governments and agencies (World Health Organization, 2020a), nurse educators at universities, colleges and hospitals are increasingly challenged to also consider innovative and creative ways of supporting both nurses and student nurses during these difficult times (Singh & Haynes, 2020). Both nurses and student nurses require ongoing personal, professional and educational supports to provide evidence‐based, safe quality care during COVID‐19. In particular, the destructive impact of COVID‐19 on nurses and student nurses well‐being and caring behaviours warrants urgent attention (Labrague & de Los Santos, 2021; Singh & Haynes, 2020).

COVID‐19 has affected the profession of nursing, highlighting an undeniable need for addressing global nurse shortages and awakening the recognition of the value of nurses in healthcare delivery, ironically in the year of the nurse 2020 (Jackson et al., 2020; World Health Organization, 2020b). However, as a profession, nursing still needs to value itself which involves recognising the importance of self‐care and promoting well‐being. Nurse education plays a critical role in cultivating an ethos of self‐care, while also empowering the nursing workforce (nurses and student nurses) to actively source and engage with personal and professional supports. Subsequently, nurse educators should critically review how they positively support clinical practice and the personal and professional development of nurses during and post‐COVID‐19.

Clinical supervision is proposed as one strategy to support and guide both nurses and student nurses to develop professionally (McCarron et al., 2017) strengthening and challenging the effectiveness of their care. Donough and van der Heever (2018) identify many benefits and complexities of clinical supervision and recommend that clinical supervision needs greater attention within undergraduate programmes. Nurses are mentors/preceptors in clinical practice with a key role in supporting quality learning environments for other nurses while also role modelling and supporting high standards of nursing care for student nurses. Therefore, investing in the supports provided to nurses as mentors/preceptors will encourage positive learning environments for staff and students, where quality standards of nursing care are reinforced and role modelled. While numerous definitions and models of clinical supervision exist, its general function and purpose is the ‘facilitation of professional support and learning to enable safe practice of healthcare professionals’ (Pollock et al., 2017, p.1826). Clinical supervision is a process of professional support and learning, through regular protected time for discussions and facilitated reflection, which can empower the supervisee to avail of opportunities to make improvements for self and patients, ultimately guiding and improving quality care delivery (Bifarin & Stonehouse, 2017; Kisthinios & Carlson, 2019; Ryan, 2021; Saab et al., 2021). Thus, clinical supervision is a worthy aspect of a required standard in clinical practice as unfortunately, past reports of sub‐standard care are evident (Francis Report, 2013; Jones et al., 2015; Keogh Report, 2013; Zelenikova et al., 2019) and a threat exists of additional reports like these during difficult time such as a pandemic or staff shortages. However, nurse educators supporting the implementation of clinical supervision offer a potential panacea against this (Markey, Murphy, et al., 2020) and educational support available in daily clinical practice can positively support both nurses and student nurses.

The nursing workforce holds an untapped capacity during this global pandemic to lead in clinical practice and clinical leadership and nurse educators need to be equally responsive in supporting their colleagues. Both formal and informal nurse leaders are to be nurtured and supported in proclaiming nursing as a strong profession (Scully, 2015). Clinical supervision can guide nurses in areas such as reflective practice, education and critical analysis of quality care provision and person‐centredness (Tomlinson, 2015). Nurse educators must also act as leaders ensuring a systematic approach to well‐being for nurses and student nurses (Singh & Haynes, 2020). This article proposes that tertiary education institutes engage with clinical partners in developing and sustaining clinical supervision as a means of supporting nurses and student nurses personal well‐being, professional development and collective resilience in responding to the complexities of COVID‐19. It examines the importance of collaboratively working to implement clinical supervision through the lens of the nursing metaparadigms, a central philosophy for nurse education and clinical practice, providing some practical suggestions as to how nurse educators can support an ethos of clinical supervision, during and post‐pandemic.

## 2 DESIGN

This is a discussion article.

## 3 METHODS

A discourse of the literature considering the importance of clinical supervision in collaboration with healthcare and educational organisations was undertaken. Approval from a Research Ethics Committee was not required for this discursive article. A scoping search of international literature pertaining to clinical supervision was conducted to provide a broad overview of the area of clinical supervision to date. The key word of ‘clinical supervision’ AND ‘nurs*’ were used in the following databases: Medline, CINAHL and PsycInfo to obtain coverage, rather than an extensive use of terms, as would be the case in focused reviews. In addition, a snowball (backward chaining) approach was used, which involved exploring references cited in relevant literature by manually searching bibliographies. General eligibility depended on peer‐reviewed literature published in English.

## 4 RESULTS

### 4.1 Clinical supervision through nursing metaparadigms

Current clinical environments are fraught with challenges for nurses and students delivering care (Martin & Snowdon, 2020). Clinical supervision is one strategy that can facilitate support for learning and empowerment through providing strategies to counteract feelings of negativity and low esteem (Cutcliffe et al., 2018; Markey, Murphy, et al., 2020). Tertiary education institutions are ideally placed to support nurses and students through supporting clinical supervision and thus helping nurses and students cope and manage in their busy hectic daily environments (Kuhne et al., 2019; Turner & Hill, 2011a). Various benefits of clinical supervision are evident from improving: the care environment (Key et al., 2019), care delivered (Esfahani et al., 2016), job satisfaction (Cutcliffe et al., 2018), development of knowledge and leadership skills (Bifarin & Stonehouse, 2017) and coping mechanisms and resilience (Gong & Buus, 2011). However, even with such benefits, there remains a low level of engagement in clinical supervision within the nursing profession (Cook et al., 2020) thus, providing an opportune time to highlight the importance of clinical supervision and promote its use and uptake.

Nursing metaparadigms provide a philosophical underpinning for practice (Parse, 2000) and were first classified by Fawcett (1978) into four categories: person, environment, health and nursing. The metaparadigms are widely used in nursing and hold relevant today as they encapsulate the essence of personal and professional practice and provide a realistic framework for nurses to map their practice and role. The person metaparadigm refers to individuals in a definite culture, family and society; the environment metaparadigm characterises all regional, national and global cultural, social, political and economic conditions related to human health; the health metaparadigm defines processes of life and death, and the nursing metaparadigm describes the nursing profession, nursing practices and nursing objectives and results (Bahramnezhad et al., 2015; Fawcett, 2000). These metaparadigms can be individually applied to clinical supervision as they allow a universal and fluid exploration of clinical supervision as a scaffolding of support for nurses and students during COVID‐19 and beyond.

### 4.2 Person

The metaparadigm of person typically focuses on the patient who is the recipient of care. In this case, the nurse or student nurse will be the recipient of the benefits of engaging with clinical supervision. Clinical supervision is ideally placed in supporting nurses in their profession and the delivery of daily caring practices, as it leads to enhancements of the capability and capacity for providing quality and safe patient care (Bifarin & Stonehouse, 2017; Esfahani et al., 2016; Key et al., 2019; Ryan, 2021). Challenges exist daily during a pandemic bringing turmoil to already stretched health services trying to deliver care (Martin & Snowdon, 2020). While nurses and student nurses remain well intended, they continue to experience a range of negative emotions when unable to provide the standard of care they expect to deliver (Maben & Bridges, 2020; Markey et al., 2019). While this challenge existed prior to the pandemic and nurses sought ways of prioritising care (O'Donnell & Andrews, 2020; Suhonen et al., 2018), this has been intensified during COVID‐19 (Martin & Snowdon, 2020). Clinical supervision is a means to raise issues within care delivery, to discuss and explore options within supervision to empower nurses to rethink care and ensure quality safe care (Cutcliffe et al., 2018; McCarron et al., 2017). Pollock et al. (2017) model of clinical supervision includes formative and normative functions, addressing education and standards of care and accountability, thus, offering a model as a framework of support for nurses and students.

Through engaging in clinical supervision nurses are enabled to develop their knowledge and competence (Kuhne et al., 2019; Snowdon et al., 2017; Turner & Hill, 2011a, 2011b), assume responsibility and increased accountability for their practice (Cutcliffe et al., 2018) and enhance consumer protection and the safety of care in complex clinical situations (Key et al., 2019; McCarron et al., 2017). Clinical supervision may provide guidance for nurses and students in promoting high standards of ethical practice and ensures the welfare of patients and staff alike (Kirwan et al., 2013) thus, can offer support in a time when difficult care decisions are required during a pandemic.

The need for preparation and training for supervisees and supervisors is well acknowledged (Hall, 2018; Key et al., 2019) and nurse educators play a critical role in designing and delivering such education initiatives in collaboration with healthcare organisations to ensure the ethos of healthcare services remains and nurses and students are not placed in conflict situations. Clinical supervisors as trained clinical staff in this role in their organisation should support, prompt and guide the supervisee to critically reflect on their practice and examine ways of improving it (Lillyman & Ghaye, 2007). Clinical supervisors need excellent facilitation, questioning, listening and reflection skills (Bifarin & Stonehouse, 2017) which nurse educators are well placed to help supervisors develop. Preparing future supervisors to support supervisees is essential and a potential suggestion may be having nurse educators become supervisors and be available to healthcare organisations to help facilitate clinical supervision sessions onsite for an interim period in supporting healthcare partners. It is noted that when supervisors are not in an active line management role of the supervisee, this is best as it reduces the risk of supervision being viewed as an evaluation (Bifarin & Stonehouse, 2017).

Within the person component of the nursing metaparadigm culture plays a central role, thus creating a culture supportive of clinical supervision as a mode of providing opportunities for staff and students to question and appraise their practices, in working towards improvements in care delivery is important (Davenport, 2013). Nurse educators, because of their unique ability to bridge the practice education gap, are positioned to influence this by providing opportunities to access and enable real‐time supervision when resources, such as time and people, are especially limited during COVID‐19 (Ducat et al., 2016).

Ideally, one‐to‐one or peer group supervision sessions over an hour duration provide the opportunity for staff and students to talk through their concerns and help develop an understanding of the situation, their actions and reactions with their supervisor. Such methods of delivery, however, are challenged during COVID‐19 times with reduced staffing levels, demands on staff, a lack of value placed on clinical supervision and limitations in the infrastructure to support alternative telecommunication meeting. They can further support staff during COVID‐19 through exploring other methods rather than face‐to‐face supervision such as online or telephone contact during COVID‐19 and beyond. Although, face‐to‐face supervision is preferred (Pinto et al., 2017), creative approaches may encourage clinical supervision uptake and accessibility. For example, online or telephone sessions may provide the answer by facilitating increased opportunities for time poor clinical environments to engage in clinical supervision (Wright & Griffiths, 2010). However, further exploration and evaluation of the impact of tele‐supervision needs to be examined to support its delivery (Martin et al., 2017). To date, no studies appear to have explored clinical supervision delivery during COVID‐19 despite the known benefits of clinical supervision and recent evidence from a survey of healthcare organisations suggests that this is still not been received by many (White & Winstanley, 2021).

Pandemic placement experiences for students enables them to experience ‘real world pressures associated with total patient care’ (Scott & Elliott, 2019, p. 43) influencing future practice (Weiss et al., 2019) and providing an essential link from theoretical content to practical skills. Many student nurses working during this pandemic report positive experiences, (Godbold et al., 2021) however, these experiences are not without challenges and many report psychological distress (Ersin & Kartal, 2020; Gómez‐Ibáñez et al., 2020; Kim et al., 2021). For those where clinical supervision is absent, support from nurse educators to establish this support mechanism is warranted where in collaboration with health service partners in times of reduced staffing nurse educators may be available to help facilitate such sessions in collaboration with clinical partners in supporting both nurses and students. Such maintenance would provide a supportive culture and society for nurses and students to reflect and learn. The inclusion of clinical supervision in undergraduate nursing education can establish clinical supervision practice at an early stage in one's nursing career and support the delivery of best standards and provide individual support in a complex time during a pandemic. Any opportunity to avail of supervision should be supported and be valued during a pandemic to help promote nurses and students' health and that of their patients.

### 4.3 Health

Safeguarding the health of nurses and student nurses is a key part of the response to this pandemic to ensure that there is a health service available to deliver care to all. The metaparadigm of health refers to the quality and wellness of the patient. In this case, the nurse or student nurse and their access to healthcare and maintaining health and well‐being. World Health Organization (2020a, 2020b) recognise that the mental health and well‐being of frontline healthcare professionals is a priority during COVID‐19. Health is in a constant state of motion and engagement with clinical supervision assists nurses and students to manage their own health and well‐being (Be´Gat & Severinsson, 2006). Nurse leaders are uniquely placed to emphasise the importance of self‐care for nurses and students' well‐being at the centre of the response to COVID‐19 (Adams & Walls, 2020; Labrague, 2021) and nurse educators supporting the delivery of clinical supervision can aid this. The health of nurses and students is always essential when caring for the sick but especially during a pandemic when they are faced daily with a deadly virus and challenged to provide care (Maben & Bridges, 2020).

Working in partnership with health service organisations, nurse educators can assist with empowering nurses to develop self‐care strategies through providing regular clinical supervision sessions for nurses and students, providing a safe space for expression of oneself, raise concerns and be guided to reflect on incidents from practice. In addition, it provides a space for both nurses and students to examine the challenges and ways of dealing with their complex nursing decisions in a supportive environment. By engaging in clinical supervision, supervisees become empowered and it provides practical experiences that will help develop supervisee's clinical supervisor role, as a means of sustaining clinical supervision.

Clinical supervision is seen to help reduce stress for nurses which for some impacts on their physical and mental health (Dhaini et al., 2017; White et al., 2019)and help reduce student stress and anxiety (Admi et al., 2018; Gurková & Zeleníková, 2018; Moked & Drach‐Zahavy, 2016). The three functions of clinical supervision: formative (developing skills and knowledge), normative (administrative, day‐to‐day matters) and restorative (supportive) support the nursing workforce, and more specifically, the restorative functions is likely to be of most help during and post‐pandemic as it can support worker mental health and well‐being (Martin & Snowdon, 2020). Through these three different functions, nurses and students can verbalise their worries and anxieties and learn of supports to help work through concerns at work which helps reduce their stress levels. Overall, practitioner well‐being is what is important in supporting nurses and students on the frontline (Moxham & Gagan, 2015) and engagement in clinical supervision can facilitate discussions for both nurses and students on their emotions in a safe and supportive environment to address stress (Dhaini et al., 2017; Towell‐Barnarda et al., 2020; White et al., 2019).

The reality is that for the nursing workforce confronted with clinical priorities and a growing clinical load, there is a tendency to prioritise patient and family welfare over one's own, sacrificing strategies aimed at one's own well‐being such as clinical supervision (Martin & Snowdon, 2020). Thus, often when support is needed most it is not sought or not available and recent literature has shown the impact COVID‐19 has had on clinical supervision for healthcare workers and students on placements and the adverse effects on clinical supervision frequency and duration (Martin et al., 2022a, 2022b; Soheilian et al., 2022).

### 4.4 Environment

Fawcett and DeSanto‐Madeya (2013) refer to the environment as a place where nursing care is delivered. Both the person and health metaparadigm can also impact on the environment as they are intrinsically linked (Fawcett, 2000). However, environmental factors influence the uptake and delivery of clinical supervision such as busy environments, lack of time and poor management (Ducat et al., 2016). Brunero and Lamont (2012) identified time away from clinical demands, availability of clinical supervisors, space and potential training costs are specific barriers to implementing clinical supervision. Thus, in the current climate, engagement in support systems such as clinical supervision is often neglected due to the pressures and demands of work (Bifarin & Stonehouse, 2017; Martin & Snowdon, 2020). However, it is more important for nurses and students while caring in a pandemic that the environment is suitable for patients and themselves. To support and care for the patient, the nurse must care for oneself and must highlight and address the environmental issues that may affect care deliver. This is compounded by the fact COVID‐19 has changed care environments adding a layer of physical restrictions and infection control measures to already complex healthcare settings (Francis & Bulman, 2019).

Clinical placements provide nursing students with the opportunity to link theory to practice, and a pandemic experience requires support from both clinical practitioners and nurse educators (Espin et al., 2021). Only through clinical supervisors being effectively supported in clinical working environment by trained clinical supervisors and access to nurse educators, can nurses and students be truly supported. Florence Nightingale identified that student nurses should be trained under the direct supervision of experienced nurses who were ‘trained to train’ (Myrick, 1998, p. 589). In the clinical environment, nurse educators can work in partnership with clinical colleagues in supporting the delivery of care through clinical supervision by acting as expert facilitators to clinical supervisors in practice. Such a support process is essential for clinical supervisors as they to need clinical supervision and the opportunity to discuss and rationalise their decision‐making and facilitation process. Having good management procedures and the support of nurse education in providing clinical supervision during these unprecedented times when complex decisions must be made is extremely important. Without such support, difficulties at work add to the work environment stress and nurses' and students strains which not only effects their health and welfare but also negatively influence care provided to patients (Koinis et al., 2015).

Environments require management to create the correct atmosphere for individual and organisational compassion and collective leadership to safeguard staff ensuring they have the right support (Bailey & West, 2020). While there may be reduced clinical supervision because of a lack of available supervisors (Ducat et al., 2016), levels of engagement in clinical supervision need to be maintained and developed rather than reduce during or following this pandemic. If anything, clinical supervision opportunities should increase as healthcare environments and staff adapt to the increasing demands on services during this pandemic, to help sustain staff in pressurised times (Maben & Bridges, 2020). Through ensuring appropriate staffing levels and resources (Recio‐Saucedo et al., 2018; Scott & Elliott, 2019) and supportive guidance (Kim et al., 2018), positive working environments can develop which consequently improve standards of care. Thus, incorporating clinical supervision can improve working environments leading to the provision of quality safe care (McCarron et al., 2017).

### 4.5 Nursing

Through tertiary educational institutions and hospitals collaboratively supporting clinical supervision, nurses and nursing students can feel appreciated and supported (Hall, 2018; O'Brien et al., 2019). Clinical supervision helps nurses and students appreciate the professional self (Dewane, 2006), appraise revise nursing performance, nursing values and beliefs, enabling one to seek learning and development which reflect improvements in quality (Key et al., 2019) and safety (Esfahani et al., 2016). This echoes the philosophy of the nursing paradigm of describing the nursing profession, nursing practices and nursing objectives and results.

Clinical supervision has a supportive function which enables nurses to raise emotional concerns about their practice and build confidence (Proctor, 1986). Engagement in clinical supervision can facilitate discussions for nurses and students on emotions in a safe and supportive space (Harvey et al., 2020) helping to address challenges identified such as compassion fatigue and burnout (Dhaini et al., 2017; White et al., 2019). Clinical supervision is reported to reduce burnout (Wallbank & Hatton, 2011) while encouraging greater resilience and the development of coping mechanisms when working in complex environments (Gong & Buus, 2011). The development of the professional through clinical supervision leads nurses and students to feel valued, appreciated and strengthen the nursing profession, especially in these challenging times (Maben & Bridges, 2020; Pollock et al., 2017). Thus, any opportunity to avail of clinical supervision should be supported and valued during a pandemic to help demonstrate compassion and support for nurses and students. The incorporation of clinical supervision, reduces burnout and increases job satisfaction and retention for staff, thus providing quality safe healthcare and improved nurse and student experiences (Cutcliffe et al., 2018; McCarron et al., 2017). These aspects relate to the nursing metaparadigm and the core values of nursing practice (care, compassion and commitment).

Clinical supervision aid's nurses in managing expectations and developing self‐awareness and coping strategies to navigate complex and busy healthcare organisations (Francis & Bulman, 2019; McCann et al., 2013), skills which were challenged in development for nurses during COVID‐19. Improved resilience can support nurses to respond to the increasing demands placed on them during this pandemic. Clinical supervision supported by clinical supervisors and nurse educators can lead the way for nurses to utilise it as a forum for learning and practice development (Dilworth et al., 2013) and critically reflect on care delivery in a supportive setting (Blishen, 2016). This opportunity supports positive professional socialisation (Bifarin & Stonehouse, 2017) which enables nurses and students to review and improve on standards of care.

The principles of clinical supervision also facilitate the development of leadership skills (Blishen, 2016), which are core requisite in today's healthcare environment. Internationally, nurses show true leadership daily, shining a light on their roles demonstrating how clinical supervision strongly influences nurses and student nurses' development of a professional identity, enhancing decision‐making ability and personal growth. Clinical supervision helps sustain and enable supervisees to flourish during times of need (Levine & Boaks, 2014). Clinical supervision is also an important support as current healthcare environments are demanding and universal signs of emotional exhaustion, fatigue and frustration are present (Maben & Bridges, 2020; Markey, Ventura, et al., 2020). Thus, clinical supervision can be the difference between success and burnout, quality patient care or extensive incidents of missed care (Markey, Murphy, et al., 2020), and thereby needs organisations support and commitment for its roll out and development.

## 5 CONCLUSION

This pandemic has demanded flexibility in every aspect of life and nurse education is no exception. With the closure of campuses internationally, nurse education had to move to the avant‐garde concept of being fully online at moments during the crisis in helping protect students and faculty. COVID‐19 has prompted nurse educators to think of how to help sustain and maintain ongoing support not only of undergraduate curriculums but that of frontline nurses and students. COVID‐19 has highlighted the importance of support measures for the nursing workforce, and this is echoed around the globe (WHOc, 2021). While support measures are being called for to better support the nursing workforce, it is essential not to overlook existing mechanisms such as clinical supervision in the support of the nursing workforce and the value of this support (Martin & Snowdon, 2020). This article discusses the necessity of clinical supervision in supporting nurses and students clinically during this pandemic and highlights the importance of clinical practitioners and educators working collaboratively to plan, develop and implement clinical supervision that can be sustained. It recognises the value of clinical supervision implementation especially during a pandemic when supports to promote engagement are reduced, yet it is the greatest hour of need for delivery in supporting burdened nurses and students. The value of clinical supervision is in its support for nurses and students while enhancing patient outcomes and the quality of nursing care and thereby an organisational approach to its development is required.

## AUTHOR CONTRIBUTIONS

All authors meet the criteria for authorship as outlined below. All entitled to authorship are listed as authors. No other authors were involved with this article. Have made substantial contributions to conception and design, or acquisition of data, or analysis and interpretation of data; (C.O'D.; K.M.; L.M.; J.T.; O.D.). Been involved in drafting the article or revising it critically for important intellectual content; (C.O'D.; K.M.; L.M.; J.T.; O.D.). Given final approval of the version to be published. Each author should have participated sufficiently in the work to take public responsibility for appropriate portions of the content; (C.O'D.; K.M.; L.M.; J.T.; O.D.). Agreed to be accountable for all aspects of the work in ensuring that questions related to the accuracy or integrity of any part of the work are appropriately investigated and resolved. (C.O'D.; K.M.; L.M.; J.T.; O.D.).

## ACKNOWLEDGEMENTS

The authors have no acknowledgements to report. Open access funding provided by IReL.

## FUNDING INFORMATION

No external funding from any funding agency in the public, commercial or not‐for‐profit sectors.

## CONFLICT OF INTEREST STATEMENT

None.

## Data Availability

Data sharing not applicable to this article as no datasets were generated or analysed during the current study.
